# Nutritional Intake and Status of Cobalamin and Folate among Non-Pregnant Women of Reproductive Age in Bhaktapur, Nepal

**DOI:** 10.3390/nu8060375

**Published:** 2016-06-22

**Authors:** Ram K. Chandyo, Manjeswori Ulak, Halvor Sommerfelt, Jørn Schneede, Per M. Ueland, Tor A. Strand

**Affiliations:** 1 Department of Community Medicine, Kathmandu Medical College, P.O. Box 21266, Sinamangal, 44621 Kathmandu, Nepal; 2 Centre for Intervention Science in Maternal and Child Health, Centre for International Health, University of Bergen, P.O. Box 7800, 5020 Bergen, Norway; halvor.sommerfelt@uib.no (H.S.); tor.strand@uib.no (T.A.S.); 3 Department of Child Health, Institute of Medicine, P.O. Box 1524, 44600 Kathmandu, Nepal; manjeswori@gmail.com; 4 Department of Clinical Pharmacology, University of Umeå, 90187 Umeå, Sweden; jorn.schneede@umu.se; 5 Institute of Medicine, University of Bergen, P.O. Box 7800, 5020 Bergen, Norway; per.ueland@ikb.uib.no; 6 Department of Research, Innlandet Hospital Trust, P.O. Box 990, 2629 Lillehammer, Norway, Lillehammer University College, 2604 Lillehammer, Norway

**Keywords:** cobalamin, folate, non-pregnant women, Nepal, methylmalonic acid, homocysteine

## Abstract

Cobalamin and folate are especially important for women of childbearing age due to their ubiquitous role in fetal growth and development. Population-based data on cobalamin and folate status are lacking from Nepal, where diets are mostly vegetarian. The objectives of the study were to investigate cobalamin and folate intake and status, and to explore associations with socio-demographics, anthropometrics, anemia, and dietary habits. Following a random selection of geographical clusters, we collected blood samples from 500 non-pregnant women and 24-h dietary recalls and food frequency questionnaires from a subsample of 379 women. Twenty percent of the women did not consume any food containing cobalamin during the days recalled, and in 72% nutritional cobalamin intake was <1 μg/day. Eighty-four percent of the women had cobalamin intake lower than the estimated average requirement (EAR) (<2 μg/day). In contrast, only 12% of the women had a folate intake less than 100 μg per day, whereas 62% had intake between 100 and 320 μg. Low plasma cobalamin (<150 pmol/L) was found in 42% of the women, most of whom (88%) also had elevated levels of methylmalonic acid. Our results indicated a high prevalence of nutritional cobalamin deficiency, while folate deficiency was uncommon.

## 1. Introduction

Deficiencies of cobalamin (vitamin B12) and folate among women of reproductive age have received increased interest not only due to associations with neural tube defects and poor cognitive development in offspring [1,2,3,4,5,6], but also because of increased risk of hyperhomocysteinemia, which may be an independent risk factor for insulin resistance and adverse pregnancy outcomes [7,8,9,10,11]. Cobalamin is a B-vitamin predominantly contained in foods of animal origin, and nutritional deficiencies have been reported in many populations where diets are predominantly vegetarian, which may lead to development of megaloblastic anemia [12,13,14].

Cobalamin is required for DNA synthesis and metabolism of folic acid, and is important for central nervous system function and most rapidly dividing cells, including maturation of red blood cells. Similarly, folate coenzymes are essential for methionine regeneration, the shuttle of one-carbon units, and RNA and DNA metabolism, as well as protein synthesis [15]. In human metabolism, periods of rapid cell division and metabolic demands, such as pregnancy and lactation, are especially vulnerable to folate and cobalamin deficiencies [16]. Methylmalonyl-CoA mutase (MM) and methionine synthase (MS) are the only two cobalamin-dependent enzymes in human metabolism. Both are essential for nervous system development in humans [7]. Cobalamin deficiency results in intra- and extracellular elevation of methylmalonic acid (MMA)—a functional marker of cobalamin status. Total homocysteine (Hcy) is remethylated to methionine by transfer of a methyl group in a reaction catalyzed by MS using 5-methyltetrahydrofolate as co-substrate. Hence, interruption of the methylation cycle resulting from deficiencies of either cobalamin or folate leads to elevation of Hcy concentration [17].

Recent clinical trials in Bangladesh and India found that cobalamin supplementation during pregnancy improved cobalamin status in both mothers and infants [18] and increased H1N1-specific IgA response in the mother [19]. Moreover, there are published reports describing the relationship between folate and cobalamin intake and functional status of these B-vitamins among women of reproductive age. Bondevik *et al.* found that 60% of pregnant Nepali women attending a hospital in Kathmandu Valley had biochemical signs of cobalamin deficiency (plasma cobalamin <150 pmol/L), while only 7% had low serum folate levels (<4.5 nmol/L) [13]. Another study among 1165 pregnant women in the first trimester residing in rural southern Nepal also found a high prevalence of cobalamin deficiency (28%), while folate deficiency was uncommon (12%) [20]. In this study cobalamin deficiency during pregnancy was also associated with an increased risk of insulin resistance in the offspring at 6–8 years of age [21]. Similarly, an increased risk of insulin resistance in children born to cobalamin-deficient women with a high folate status was also observed in a study from Pune, India [22].

Metabolic or hormonal changes and hemodilution might be limiting factors when trying to assess the prevalence of cobalamin and folate deficiency during pregnancy based on serum or plasma levels of these vitamins [23,24]. Moreover, clinical or subclinical infections are widespread in low- and middle-income countries and may confound biomarkers of folate and cobalamin status [25]. In the present study, we simultaneously explored the status and nutritional intake of cobalamin and folate and tried to identify determinants of vitamin status in a random sample of healthy, non-pregnant women residing in the semi-urban municipality of Bhaktapur, Nepal.

## 2. Materials and Methods

### 2.1. Study Site and Population

Kathmandu valley includes the districts of Kathmandu, Lalitpur, and Bhaktapur. Bhaktapur municipality, the study site, has a population of approximately 80,000, and the majority of people have agriculture as their main occupation [26]. The study site includes a semi-urban area with a large proportion of the population having a low income. Although not continuous, drinking water is mostly from a piped government supply and most households have toilets connected with a central sewage system; wastewater exits without treatment in a nearby river. The indigenous populations of Bhaktapur municipality are the Hindu/Buddhist Newars. Bhaktapur is one of the most densely populated among the 75 districts of Nepal. Around the city of Bhaktapur, there are many carpet factories in which migrant families live and work. A factory may be inhabited by many families who share a kitchen and toilets; most of these consist of young couples and their children. They belong to different ethnic groups and come from various regions in Nepal, and income is very much dependent on the work load at carpet factories. So their food intake is accordingly highly dependent on their daily income.

### 2.2. Methods and Study Design

We used a cluster stratified random sampling procedure. We defined two strata, one stratum of local resident women and the other of those residing in the carpet factories. Clusters were either neighborhood streets (“Toles”) or carpet factories. A total of 23 Toles and five carpet factories were randomly chosen from the municipality as primary sampling units. The likelihood of selecting a cluster was made proportional to the eligible population in the particular Tole or carpet factory. Details on study design and recruitment procedures were published elsewhere [27,28].

Briefly, we obtained a list of all 2736 women between 13 and 35 years living in these selected clusters during September 2000 to November 2001. Two 24-h dietary recalls were obtained from each woman, approximately one week apart. The women were asked to recall all food consumed on the previous day from waking up until the next morning, thus covering a 24-h period. The field workers were trained to undertake the dietary recall interview before the start of the study. The first recall was carried out in the clinic and screened by a study physician for any ongoing or chronic illnesses, whereas for the second recall the field workers visited each participating woman in her home. For the food frequency questionnaire (FFQ), which was completed by 394 women, a total of 53 commonly available local foods were listed and the women were asked how often they had consumed them during the last six months. Models of commonly used local foods were shown during the interviews to estimate the portion sizes. The dietary recalls were done on weekdays as well as on weekends and during minor festivals. However, the 24-h dietary recalls were not done during major local festivals like Dashain and Tihar, or if the women had been fasting the day before the recall.

### 2.3. Ethical Approval

The Institutional Review Board at Institute of Medicine, TU, Nepal (IOM-59-00) and the Norwegian regional health authorities of the University of Bergen approved the study. The implementation of all aspects of the project was in agreement with the International Ethical Guidelines for Research Involving Human subjects as stated in the latest version of the Helsinki Declaration. Iron and folic acid were given to all enrolled subjects with anemia, according to national guidelines. Participation was voluntary, and at any time the women could withdraw consent without giving any reason. All women were offered treatment with Albendazole for intestinal parasites (if the woman had not received any dose during the past year), multivitamin supplementation, and examination by a gynecologist when indicated. Albendazole is an anti-parasitic drug not absorbed in the gut, and does not affect the bacteria used in our vitamin assays. Thus, if a woman had received Albendazole it is very unlikely that it could have affected the vitamin concentrations.

### 2.4. Blood Sampling and Processing

We collected blood from the cubital vein into micronutrient-free heparinized polypropylene tubes (Sarstedt, Germany) at least 2 h after a meal. The hemoglobin concentration was analyzed immediately following blood sampling with Hemocue (Ångelholm, Sweden). The heparinized blood was centrifuged for 10 min at 2000–2500 rpm within 10 min after venipuncture. The times of the last meal, blood collection, and plasma separation were recorded. Plasma was separated, transferred into micronutrient-free polypropylene vials (Eppendorf, Germany), and stored at −20 °C in Nepal until transfer to Norway. The specimens were transported to Norway on ice packs or with dry ice and stored at −70 °C for 0–9 months before analysis. Plasma Hcy and MMA were analyzed using the gas chromatography–mass spectrometry (GC-MS) method adapted from Husek *et al.* based on ethylchloroformate derivatization [29]. Both analyses are enrolled in an external quality control program on a regular basis [30]. The between-day coefficient of variation was <2%–3% for both analyses. The plasma concentrations of folate and cobalamin were determined using microbiological assays [31,32] where we used folic acid as a calibrator adapted to a micro titer plate format and carried out by a robotic workstation (Microlab AT plus 2; Hamilton Bonaduz AG, Bonaduz, Switzerland) [33].

### 2.5. Statistical Analysis

The data were double entered into a Microsoft Visual FoxPro database with computerized logic, range, and consistency checks. The daily intake of the various nutrients was calculated using Indian and Nepali food tables from Wfood2 [34]. The total cobalamin and folate contributions from the different foods were derived from the nutritive values of the 24-h dietary recalls, whereas the frequency of reported consumption was derived from the FFQs. Based on the 24-h dietary recalls, we present the proportion with low mean intake of energy, folate, and vitamin B12. We also adjusted the distribution of the observed intakes based on the within-person variability. Such adjustment was not possible for vitamin B12 because the intake was extremely right-skewed with 257 days without any recorded intake at all. This adjustment was done using the IMAPP software [35].

Statistical analyses were undertaken using Stata^®^, Version 10 (STATA Corp, Houston, TX, USA). Means, medians, interquartile ranges, standard deviations, and 95% confidence intervals (CI) were computed and normality was checked by visual display in scatter plot or histogram. Two-sample t-tests for continuous variables and chi-square tests for dichotomous variables were used to compare differences between carpet workers and local residents. The Spearman rank-order correlation coefficient was used to assess the relations between relevant biochemical markers and intake of cobalamin and folate; *p*-values < 0.05 were considered statistically significant. The data were analyzed using the design-based inference method, which adjusts for the cluster sampling in the survey. We used plasma cobalamin (*p-*cobalamin), folate, Hcy, and MMA levels as the dependent outcome variables in a hierarchical multiple linear regression model. Potential confounding variables in the final model were selected based on their unadjusted association with the dependent variables with a *p*-value of < 0.2. The final regression model was developed by backward selection procedure and consisted of socio-demographic features (age, working in carpet factories, literacy) and body mass index (BMI) [36]. The graphs describing the relation between p-cobalamin and plasma Hcy, MMA, and plasma folate with Hcy and folate intake were constructed using two-way fractional polynomial prediction plots in the Stata program.

### 2.6. Definitions

Anemia was defined as a hemoglobin concentration <12.3 g/dL. This cutoff was adjusted for the altitude of Kathmandu Valley (1400 m) by adding 0.3g/dL to the standard value [37]. The cutoff values for low cobalamin, low folate, and high MMA were <150 pmol/L, <6.8 nmol/L, and >0.26 μmol/L, respectively [38,39]. A commonly used cutoff value of Hcy is >15 μmol/L for non-pregnant women, which is mainly used in studies of the elderly [40]. This cutoff may be too high for our population of young, healthy females. As there is not a well-established cutoff value for Hcy, in non-pregnant women of reproductive age we divided the Hcy values into four categories: ≤7.5, 7.6–10, 10.1–15, and >15 μmol/L.

We compared the cobalamin and folate intake among the women with the recommended dietary allowances (RDA) of World Health Organization (WHO) and Food and Agricultural Organization (FAO), which are 2.4 μg and 400 μg, respectively [41]. The corresponding estimated average requirement (EAR) value of cobalamin is 2 μg and 320 μg for folate. We also assessed the RDA of the Indian Council of Medical Research, which is 1 μg for cobalamin and 200 μg for folate [42].

The frequencies of food consumption from FFQs were categorized into <1 time/month or never, 1–3 times/month, and once a week or more often. The BMI is a useful measure of malnutrition in adults. We used a cutoff value of 18.5 kg/m^2^ (WHO classification of BMI for non-pregnant women) to define chronic energy deficiency.

## 3. Results

### 3.1. General Characteristics

**Figure 1:**
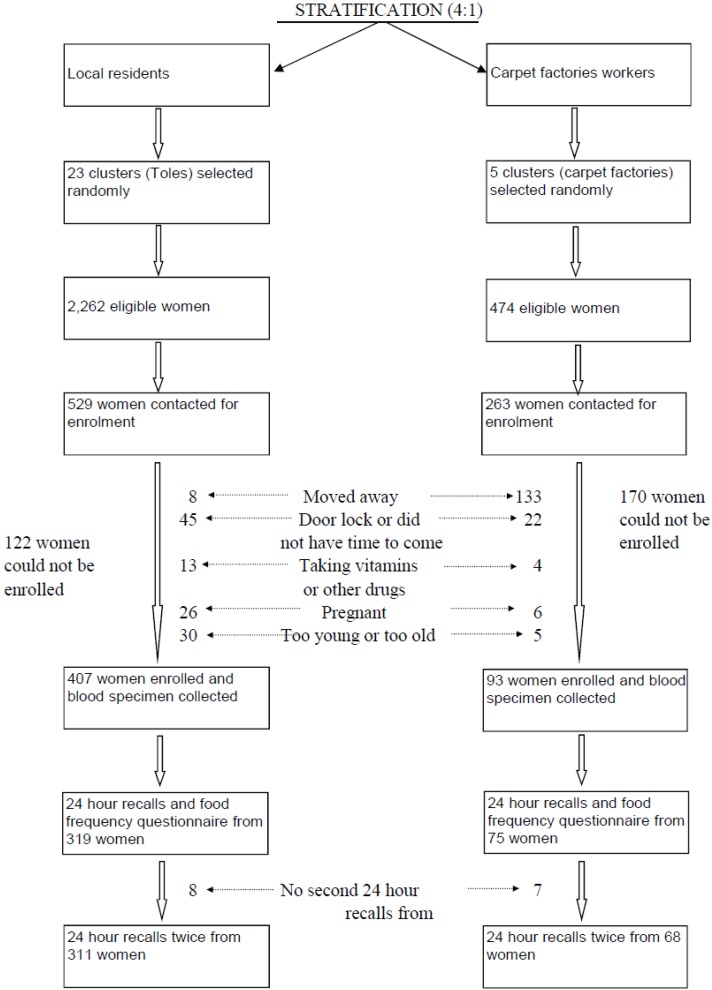
Selection procedure and study profile of a study evaluating cobalamin and folate status in Bhaktapur, Nepal.

Seven hundred and ninety-two women were randomly selected, proportional to the population size of each selected cluster (Figure 1). Two hundred and ninety-two women whom we approached could not be enrolled, mainly because they had moved (141 women) or did not have time to come to the study clinic (67 women) for enrollment. Most of the women who had moved away were from the carpet factories (133 women). We undertook FFQs from 394 women and two dietary recalls in 379 of the 500 women. The analysis of nutrient intake was restricted to these 379 women, using the mean intake of the two recalls. The participation rate in this study is more than 90%.

The mean age of the 500 enrolled women (97 from the carpet factory stratum) was 23 years and 34% were <20 years old. Fifty-two percent of the women lived in joint families, and the mean family size was seven. The mean BMI was 21.8 kg/m^2^ (standard deviation [SD] 3.0), with 11% having a BMI of <18.5 kg/m^2^ and 13% a BMI of >25 kg/m^2^ indicating chronic energy deficiency and overweight, respectively (Table 1). Two thirds of women said that they could read and write, whereas 89% of their husbands reported the same. Most of the women (49%) were working on a daily wage basis with an average of 5.8 h per day spent outside the home. Fifty-nine percent of the women were married, and among these, 74% were using contraceptives, mainly Depo-Provera progesterone injections (46%) and permanent sterilization (18%) of one of the spouses. Overall, 28% families were living in a single room but this figure was three times higher among carpet factory workers. Very few women were vegetarians (2%) in our population, but only 40% reported consumption of meat at least once a week during the last six months. Forty-six percent of the women were nullipara, 13% were para 1, 24% were para 2, and 17% were para 3 or more. The mean hemoglobin level was 13.2 g/dL (SD 1.2) and the prevalence of anemia (hemoglobin <12.3 g/dL) was 16%.

### 3.2. Cobalamin Intake

Among the 758 dietary recalls from the 379 women, the median intake of cobalamin was 0.34 μg (interquartile range [IQR]; 0.08, 1.1). The cobalamin intake varied according to age. Median intake was 0.25 μg (IQR; 0.06, 0.6) among women younger than 20, while it was 0.42 μg (IQR; 0.08, 1.3) in women ≥20 years (*p* = 0.02). Twenty percent of the women did not consume any cobalamin during the days recalled, and about two thirds (72%) consumed cobalamin <1 μg/day. Eighty-four percent of the women had cobalamin intakes less than the EAR (<2 μg/day) (Table 2). The distribution of cobalamin intake was skewed to the left with very few women consuming adequate amounts.

Buffalo meat, milk, and eggs were the foods that contributed most to the cobalamin supply. Analysis of the 24-h dietary recalls showed that buffalo meat provided 58% of the cobalamin intake in this population and it was recorded in 18% of the recalls (Table 3). Milk, separately or with tea, contributed to 23% of the cobalamin intake, and was recorded in 61% of the recalls.

Based on the FFQs, 40% of the women reported buffalo meat consumption at least once a week, 51% reported intake of once a month or less, and 9% reported they had not eaten buffalo meat within the past six months. Sixty-three percent reported that they had tea with milk at least once a week; among them, 41% reported at least once daily. Twenty-two percent had tea with milk once a month or less, while 15% reported not having consumed tea with milk during the last six months. One third of the women reported consumption of eggs at least once per week, while 60% consumed eggs once a month or less, and 7% had not consumed any eggs during the last six months.

### 3.3. Folate Intake

The median intake of folate was 211 μg (IQR; 130, 327). Green leafy vegetables like spinach, mustard, radish leaves, and dry leafy vegetables contributed to 54% of the total folate intake. Rice was recorded in all recalls, while 40% of the recalls reported different seasonal green leafy vegetables. Pulses or legumes were recorded in 61% of the 24-h dietary recalls. Only 12% of the women had a folate intake less than 100 μg per day, whereas 62% had intake between 100 and 320 μg. We also estimated the proportion with low intake adjusting for within-subject variability. With this adjustment, 69% had an intake lower than 320 µg/day. Folate intake during the monsoon season (May–August) was low, with a mean intake of 199 μg (SD 129) as compared with 262 μg (SD 124) in other seasons (*p* ≤ 0.0005), probably reflecting the seasonal availability of different green vegetables in the local community. Similar seasonal differences were also observed in the p-folate level (15.2 nmol/L during monsoon time *vs.* 26.2 nmol/L during other seasons, *p* ≤ 0.0005). Among the 394 women who completed the FFQs, 81% reported consumption of different types of green leafy vegetables, and 27% reported consuming dried vegetables at least once a week within the past six months. Sixty-one percent of the women reported consumption of pulses, and 65% had wheat flour bread at least once a week during the past six months.

### 3.4. Plasma Cobalamin, Folate, MMA, and Hcyβ

**Figure 2:**
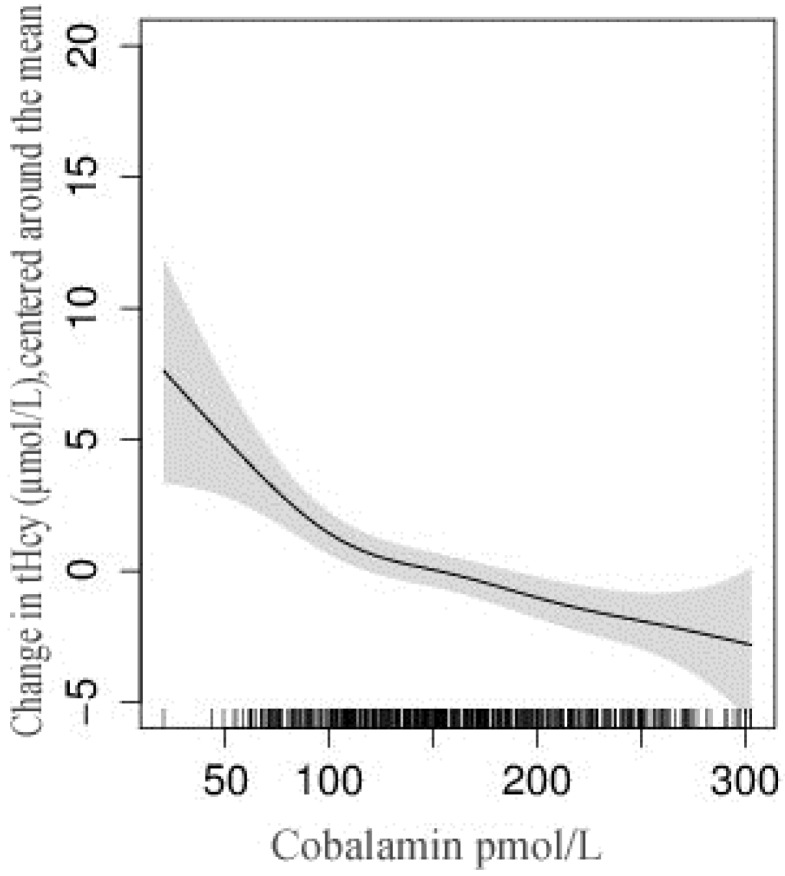
Relation between total homocysteine (tHcy) and plasma cobalamin concentration among non-pregnant women in Bhaktapur, Nepal. The vertical lines on the x-axis represent the number of subjects and the shaded area represents 95% confidence interval (CI) of the homocysteine concentration.

**Figure 3:**
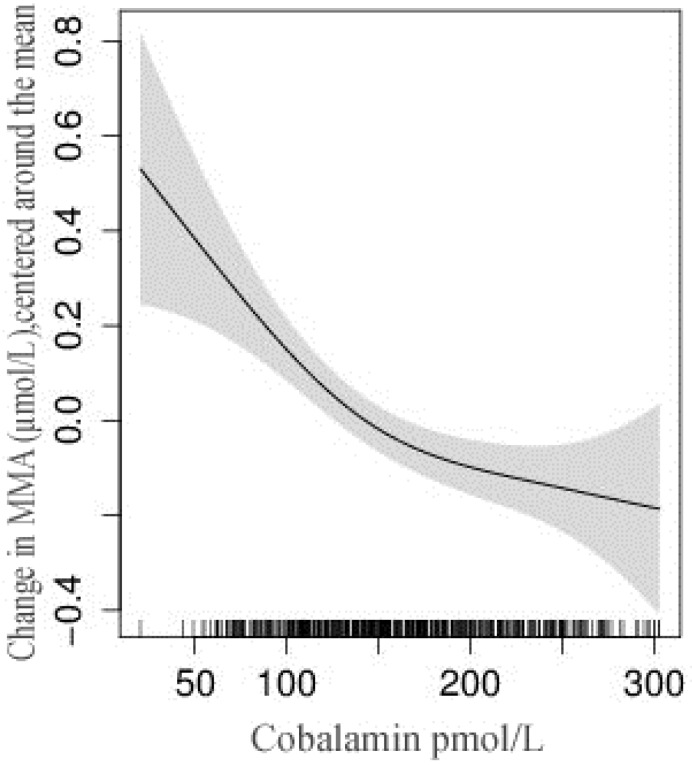
Relationship between methylamalonic acid and plasma cobalamin concentration among non-pregnant women in Bhaktapur, Nepal. The vertical lines on the x-axis represent the number of subjects and the shaded area represents 95% confidence interval (CI) of the methylmalonic acid (MMA) concentration.

Four of the 500 women had missing MMA and Hcy results, while one had missing values for p-folate and cobalamin results. The mean (SD) of Hcy, MMA, cobalamin, and folate were 10.5 μmol/L (6.1), 0.62 μmol/L (0.58), 206.0 pmol/L (188.1), and 22.4 nmol/L (17.1), respectively. 82% had elevated MMA levels (>0.26 μmol/L), while 72% had Hcy >7.5 μmol/L (Table 2). Hcy values >10 μmol/L were observed in 36% of women, while only 10% had Hcy values >15 μmol/L. Forty-two percent of the women had low cobalamin (<150 pmol/L), while only 5% had low p-folate (<6.8 nmol/L). These figures were 26% and 24% among the carpet workers. Thirteen percent of women had plasma cobalamin >250 pmol/L, which indicates adequate cobalamin status and a similar proportion of women having plasma cobalamin <100 pmol/L. Among the women with Hcy >7.5 μmol/L, 87% had elevated MMA or low cobalamin levels. The relation between Hcy and MMA with plasma cobalamin is depicted in Figure 2 and Figure 3. The combination of low *p*-cobalamin and high MMA was found among 185 women (37%), indicating most of the women with low *p*-cobalamin also had a higher MMA concentration. Spearman’s rank-order correlation showed a strong positive association between Hcy and MMA (*r* = 0.27), but no associations with cobalamin and folate levels (*r* = −0.01). The plasma MMA concentration was strongly and negatively associated with *p*-cobalamin (*r* = −0.37), but only weakly with the p-folate levels (*r* = −0.02).

Multiple linear regression analyses (Table 4) indicated that carpet workers older than 20 and women who consumed meat at least once a week had significantly higher *p*-cobalamin concentrations. P-folate was, however, significantly lower among these women, though marginally significant among women who reported consuming meat at least once a week. Plasma MMA was significantly higher among women younger than 20 and illiterate women. Surprisingly, none of the biochemical parameters of cobalamin and folate were significantly associated with anemia.

### 3.5. Relationship between Intake and Status of Cobalamin and Folate

Intake of cobalamin adjusted for dietary energy, as determined by the 24-h dietary recall method, was strongly and positively correlated with the *p*-cobalamin level (*r* = 0.18, *p* = 0.001), and negatively associated with MMA (*r* = −0.27, *p* = 0.04). A similar relationship was also observed with the folate intake and p-folate. Neither cobalamin nor folate intake was significantly associated with Hcy.

## 4. Discussion

In this cross-sectional study of healthy young non-pregnant women from a semi-urban community, we found a high prevalence of cobalamin deficiency as defined by plasma cobalamin <150 pmol/L, but only a few women were folate deficient. The results from the dietary recalls support our findings, as the most frequently consumed foods contained high levels of folate but low amounts of cobalamin. There are limited data on cobalamin and folate status in representative samples from low- and middle-income countries. Moreover, few studies include both biochemical analyses and dietary recall assessment of vitamin deficiencies. The available data on biochemical markers of folate and cobalamin status among women are mainly based on elderly or pregnant women [20,39] and none have deliberately focused on fertile women. Despite being carried out more than a decade ago, we believe that our findings are still relevant in order to document the status of cobalamin and folate along with its metabolites. Similar findings of mild cobalamin deficiency have been reported for Nepalese children [43] and pregnant women [13,20] among Indian populations [12,37,44], and among Asians living in the United States [45]. The mean folate level in our study was 22.4 nmol/L, which is higher than among Taiwanese vegetarians [46] and omnivorous Indians [39]. The mean Hcy concentrations in the present study are comparable with those reported in the study by Bondevik *et al.* [13] but lower than studies among Indians [39,44].

We compared our dietary intake of cobalamin and folate with the dietary reference intake values from the WHO/FAO [41]. As Nepalese food tables are not yet fully developed, we used Indian food tables for most of the food items, which is one of the limitations of our study. Our finding of 11% underweight *versus* 76% consuming <2200 Kcal also indicate possibilities of underreporting food items. Or it could be partly because we did not require dietary recall during major local festival and celebrations. Using the Indian RDA, which is 1 μg/day for cobalamin and 200 μg/day for folate [42], 72% of the women would have a lower than recommended intake of cobalamin, whereas 47% had a lower than recommended intake of folate. We did not have data on creatinine, as a measure of reduced kidney function, which may influence MMA and total homocysteine concentrations [47]. However, as we enrolled apparently healthy young women, it is reasonable to assume that most of the presumed healthy subjects had normal kidney function.

The poor cobalamin status in our population may partly be explained by the traditional predominantly vegetarian diet in Nepal. Among the 775 24-h dietary recalls, only 18% reported any meat intake, which indicates that even though the majority of women were not vegetarian, meat consumption is not regular. More than half of the study population reported that they had only consumed meat 1–3 times a month or less during the past six months. Consumption of meat (buffalo) at least once a week was strongly associated with improved cobalamin status. This could be one of the reasons for our finding of relatively better cobalamin status among women from carpet factories, as 59% of them reported at least weekly meat consumption compared to 36% among the local resident women. Buffalo meat constitutes about two thirds of the total meat consumption and carpet workers, in contrast to the indigenous population, also consume pork meat [48]. Non-dietary contribution of cobalamin through synthesis by intestinal microflora [49] could also be possible among carpet women as most of them live in poor conditions with shared rooms and toilet facilities. Although one third of the included women reported that they consumed eggs at least once a week, only 9% of the total 24-h dietary recalls reported on this particular food and a recent study indicated that egg intake did not significantly contribute to higher plasma cobalamin concentrations compared to meat or diary intake [50].

Fish is a rich source of cobalamin (2.32 μg per 100 g), but less than 1% of the recalls included any fish. Fish and yogurt, however, were two foods that were associated with improved cobalamin status among urban South Indian pregnant women [12]. The cobalamin content in animal milk is on average 0.46 μg per 100 g and contributed to 23% of the cobalamin intake in this population due to its frequent consumption with tea. About 60% of the recalls contained milk separately or with tea, and 41% reported consumption of tea with milk once daily in the last six months.

Folic acid supplementation is recommended primarily to prevent neural tube defects and is effective if given during the periconceptional period [51]. In Nepal, the majority of pregnant women make the first antenatal visit after completion of the first trimester [52]. Folic acid food fortification has been suggested in many countries to prevent neural tube defects [53]. However, due to the “folate trap” mechanism, isolated folic acid supplementation might be harmful in people with latent cobalamin deficiency [54,55]. MMA, Hcy, folate, and cobalamin were not associated with anemia in our study. A similar lack of associations between marker of B12 status and anemia has also been reported in other populations, such as among pregnant Nepali women [13], among Indians [39], and in the Swedish elderly [56]. Most of the elevated Hcy in our study was explained by low plasma cobalamin concentrations, which are probably due to the adequate concentration of folate in this population.

## 5. Conclusions

Results of blood samples and dietary intake recalls from representative women indicated a high prevalence of cobalamin deficiency, while folate deficiency was uncommon. It is important to investigate the potential negative effects of cobalamin deficiency in this population, and further research should focus on the health consequences of elevated plasma Hcy and impaired cobalamin status in Nepal. Prospective studies in representative populations identifying dietary and other attributable risks of cobalamin deficiencies and elevated Hcy are warranted.

## Figures and Tables

**Table 1:** General characteristics of 500 women included in a study evaluating cobalamin and folate status in Bhaktapur, Nepal.

Characteristics	% (n)
Mean age (SD), y	23 (6)
Parity one or more, n (%) ^1^	54 (268)
Mean age of menarche (SD), y	14.6 (1.5)
Number of women using contraceptives ^2^	74 (219)
Number of women who smokes ^3^	6.6 (26)
Number of vegetarians ^3^	2 (10)
Mean family size (SD)	7 (3)
Number of women working only in agriculture	23 (117)
Number of women working on daily wage basis	49 (247)
Mean body mass index, BMI (SD), kg/m^2^	21.8 (3.0)
Number of women with BMI <18.5 kg/m^2^	11 (55)
Mean Hemoglobin (SD), g/dL	13.2 (1.2)
Total number of women with Hb <12.3 g/dL	16 (79)
Number of women who cannot read/write	33 (166)

^1^ % (n) all such data unless otherwise indicated; ^2^ Among 296 married women using temporary or permanent family planning; ^3^ Data from 394 women from whom we collected food frequency questionnaires.

**Table 2:** Intakes of energy, cobalamin and folate and plasma levels of homocysteine, methylmalonic acid, cobalamin and folate among non-pregnant women in Bhaktapur, Nepal.

Indicators	Values
Dietary intake (*n* = 379)	
Energy, kcal (median, IQR)	1894 (1576, 2184)
% consuming <2200 Kcal, % (n) ^1^	76 (289)
Folate, µg (median, IQR)	211 (130, 327)
% consuming <320 µg	74 (279)
% consuming <200 µg	47 (178)
Cobalamin, µg (median, IQR)	0.34 (0.08, 1.1)
% consuming <2 µg	88 (333)
% consuming <1 µg	72 (271)
Biochemical markers (*n* = 500)	
Homocysteine (Hcy), µmol/L, mean (SD)	9.0 (7.4, 11.4)
% of Hcy >7.5 µmol/L	72 (361)
% of Hcy >10 µmol/L	36 (182)
% of Hcy >15 µmol/L	10 (52)
Folate, nmol/L (mean,SD)	22.4 (17.1)
% of plasma folate <6.8 nmol/L	4.6 (23)
% of plasma folate <10 nmol/L & Hcy >7.5 µmol/L	4 (22)
Methylmalonic acid (MMA), µmol/L (mean, SD)	0.62 (0.58)
% of MMA >0.26 µmol/L	82 (405)
Cobalamin, pmol/L (mean,SD)	173.2 (74.4)
% of plasma cobalamin <150 pmol/L	42 (210)
% of plasma cobalamin <150 pmol/L & MMA >0.26 µmol/L	37 (185)
% of plasma cobalamin <150 pmol/L & Hcy >7.5 µmol/L	34 (170)
% of plasma cobalamin <150 pmol/L & plasma folate <6.8 nmol/L	2 (10)

^1^ % (n)—all such values unless otherwise indicated.

**Table 3:** Main food sources of cobalamin and folate among non-pregnant women in Bhaktapur, Nepal ^1^.

Frequency of Consumption				
Foods	Contribution (%)	Number of Recalls with Particular Food (%)	Number of Women Who Reported to Consumption Atleast a Week ^2^ % (n)	Content (µg) Per 100 gr Food ^3^
**Cobalamin**				
Buffalo meat	58%	18	40 (159)	1.47
Milk (buffalo) ^4^	23%	61	63 (247)	0.46
Eggs	9%	9	33 (130)	1.1
Goat meat	4%	1	1 (4)	1.52
Curds (yogurt)	4%	4	3 (12)	0.37
Fish	1%	0.5	1 (4)	2.32
Poultry	1%	3	7 (28)	0.25
**Folate**				
Green or dry leafy	54%	57	81 (319)	118–146
vegetables				
Rice	8%	100	100 (394)	5.8
Pulses/Beans	7%	61	67 (264)	182
(lentil, gram, peas)				
Wheat flour or grain	5%	41	65 (256)	30
Potato	4%	73	88 (347)	9
Rice flakes	2%	33	33 (130)	12

^1^ Data based on 379 women from whom we obtained two 24 h dietary recalls; ^2^ Data from food frequency questionnaire; ^3^ Data from Indian food table of wfood2 program; ^4^ Consumption of milk separately or with tea.

**Table 4:** Multiple linear regression for the association of age, body mass index and socio-demographic features with plasma cobalamin, folate, methylmalonic acid and homocysteine among women in Bhaktapur, Nepal.

Variables	n (%)	Plasma Cobalamin	Plasma Folate	Plasma Methylmalonic Acid	Plasma Homocystiene
Age <20 years	500				
No	328 (65.5)				
Yes	172 (34.4)	(−18.5; −32.8, −4.2) ^1^	(−4.2; −7.6, −0.88)	(0.15; 0.04, 0.27)	(0.87; −0.36, 2.1)
Working in carpet factories	500				
No	407 (81.4)				
Yes	93 (18.6)	(37.5; 19.6, 55.4)	(−4.5; −8.7, −0.34)	(−0.11; −0.25, 0.03)	(−0.05; −1.6. 1.5)
Illiterate mother	497				
No	331 (66.6)				
Yes	166 (33.4)	(4.5; −10.5, 19.5)	(−0.53; −4.1, 3.0)	(0.15; 0.03, 0.27)	(−0.52, −1.8, 0.76)
BMI <18.5 kg/m^2^	500				
No	445 (89)				
Yes	55 (11)	(3.2; −17.5, 23.9)	(−1.7; −6.6, 3.1)	(0.03; −0.13, 0.19)	(0.23; −1.5, 1.9)
Atleat once a week meat intake ^2^	394				
No	234 (59.5)				
Yes	160 (40.5)	(30.5; 15.8, 45.1)	(−0.36; −4.1, 3.4)	(−0.19; −0.3, −0.09)	(0.43; −1.7, 0.89)
Anemia (Hb <12.3 g/dL)	500				
No	421 (84.2)				
Yes	79 (15.8)	(6.4; −12.1, 24.9)	(0.42; −3.9, 4.8)	(−0.11; −0.25, 0.04)	(−0.45; −2.0, 1.1)

^1^ Adjusted regression coefficient and *95% CI* obtained from linear regression model adjusted for the variables included in this table; ^2^ Intake of buffalo meat was based on 394 women from whom we collected food frequency questionnaires.

## Abbreviations

The following abbreviations are used in this manuscript:

MMA	methylmalonic acid
Hcy	plasma total homocysteine
FFQ	food frequency questionnaire
RDA	Recommended Dietary Allowances

## References

[1] Dror D.K., Allen L.H. (2008). Effect of vitamin b12 deficiency on neurodevelopment in infants: Current knowledge and possible mechanisms. Nutr. Rev..

[2] Stiles J., Jernigan T.L. (2010). The basics of brain development. Neuropsychol. Rev..

[3] Molloy A.M., Kirke P.N., Troendle J.F., Burke H., Sutton M., Brody L.C., Scott J.M., Mills J.L. (2009). Maternal vitamin B12 status and risk of neural tube defects in a population with high neural tube defect prevalence and no folic acid fortification. Pediatrics.

[4] Bhate V., Deshpande S., Bhat D., Joshi N., Ladkat R., Watve S., Fall C., de Jager C.A., Refsum H., Yajnik C. (2008). Vitamin B12 status of pregnant Indian women and cognitive function in their 9-year-old children. Food Nutr. Bull..

[5] Veena S.R., Krishnaveni G.V., Srinivasan K., Wills A.K., Muthayya S., Kurpad A.V., Yajnik C.S., Fall C.H. (2010). Higher maternal plasma folate but not vitamin B-12 concentrations during pregnancy are associated with better cognitive function scores in 9- to 10- year-old children in South India. J. Nutr..

[6] Muthayya S., Kurpad A.V., Duggan C.P., Bosch R.J., Dwarkanath P., Mhaskar A., Mhaskar R., Thomas A., Vaz M., Bhat S. (2006). Low maternal vitamin b12 status is associated with intrauterine growth retardation in urban South Indians. Eur. J. Clin. Nutr..

[7] Stabler S.P. (2013). Vitamin B12 deficiency. N. Engl. J. Med..

[8] Ueland P.M., Refsum H., Beresford S.A., Vollset S.E. (2000). The controversy over homocysteine and cardiovascular risk. Am. J. Clin. Nutr..

[9] Nelen W.L., Blom H.J., Steegers E.A., den Heijer M., Thomas C.M., Eskes T.K. (2000). Homocysteine and folate levels as risk factors for recurrent early pregnancy loss. Obstet. Gynecol..

[10] Eskes T.K. (2001). Clotting disorders and placental abruption: Homocysteine—A new risk factor. Eur. J. Obstet. Gynecol. Reprod. Biol..

[11] Hogeveen M., Blom H.J., den Heijer M. (2012). Maternal homocysteine and small-for-gestational-age offspring: Systematic review and meta-analysis. Am. J. Clin. Nutr..

[12] Samuel T.M., Duggan C., Thomas T., Bosch R., Rajendran R., Virtanen S.M., Srinivasan K., Kurpad A.V. (2013). Vitamin B(12) intake and status in early pregnancy among urban south indian women. Ann. Nutr. Metab..

[13] Bondevik G.T., Schneede J., Refsum H., Lie R.T., Ulstein M., Kvale G. (2001). Homocysteine and methylmalonic acid levels in pregnant nepali women. Should cobalamin supplementation be considered?. Eur. J. Clin. Nutr..

[14] Herbert V. (1994). Staging vitamin B-12 (cobalamin) status in vegetarians. Am. J. Clin. Nutr..

[15] Varela-Moreiras G., Murphy M.M., Scott J.M. (2009). Cobalamin, folic acid, and homocysteine. Nutr. Rev..

[16] Casterline J.E., Allen L.H., Ruel M.T. (1997). Vitamin B-12 deficiency is very prevalent in lactating Guatemalan women and their infants at three months postpartum. J. Nutr..

[17] Barbosa P.R., Stabler S.P., Machado A.L., Braga R.C., Hirata R.D., Hirata M.H., Sampaio-Neto L.F., Allen R.H., Guerra-Shinohara E.M. (2008). Association between decreased vitamin levels and MTHFR, MTR and MTRR gene polymorphisms as determinants for elevated total homocysteine concentrations in pregnant women. Eur. J. Clin. Nutr..

[18] Duggan C., Srinivasan K., Thomas T., Samuel T., Rajendran R., Muthayya S., Finkelstein J.L., Lukose A., Fawzi W., Allen L.H. (2014). Vitamin B-12 supplementation during pregnancy and early lactation increases maternal, breast milk, and infant measures of vitamin B-12 status. J. Nutr..

[19] Siddiqua T.J., Ahmad S.M., Ahsan K.B., Rashid M., Roy A., Rahman S.M., Shahab-Ferdows S., Hampel D., Ahmed T., Allen L.H. (2016). Vitamin B12 supplementation during pregnancy and postpartum improves B12 status of both mothers and infants but vaccine response in mothers only: A randomized clinical trial in bangladesh. Eur. J. Nutr..

[20] Jiang T., Christian P., Khatry S.K., Wu L., West K.P. (2005). Micronutrient deficiencies in early pregnancy are common, concurrent, and vary by season among rural Nepali pregnant women. J. Nutr..

[21] Stewart C.P., Christian P., Schulze K.J., Arguello M., LeClerq S.C., Khatry S.K., West K.P. (2011). Low maternal vitamin B-12 status is associated with offspring insulin resistance regardless of antenatal micronutrient supplementation in rural Nepal. J. Nutr..

[22] Yajnik C.S., Deshpande S.S., Jackson A.A., Refsum H., Rao S., Fisher D.J., Bhat D.S., Naik S.S., Coyaji K.J., Joglekar C.V. (2008). Vitamin B12 and folate concentrations during pregnancy and insulin resistance in the offspring: The pune maternal nutrition study. Diabetologia.

[23] Milman N., Byg K.E., Bergholt T., Eriksen L., Hvas A.M. (2006). Cobalamin status during normal pregnancy and postpartum: A longitudinal study comprising 406 Danish women. Eur. J. Haematol..

[24] Murphy M.M., Fernandez-Ballart J.D. (2011). Homocysteine in pregnancy. Adv. Clin. Chem..

[25] Abbenhardt C., Miller J.W., Song X., Brown E.C., Cheng T.Y., Wener M.H., Zheng Y., Toriola A.T., Neuhouser M.L., Beresford S.A. (2014). Biomarkers of one-carbon metabolism are associated with biomarkers of inflammation in women. J. Nutr..

[26] CBS (2011). National Population and Housing Census.

[27] Chandyo R.K., Strand T.A., Mathisen M., Ulak M., Adhikari R.K., Bolann B.J., Sommerfelt H. (2009). Zinc deficiency is common among healthy women of reproductive age in Bhaktapur, Nepal. J. Nutr..

[28] Chandyo R.K., Strand T.A., Ulvik R.J., Adhikari R.K., Ulak M., Dixit H., Sommerfelt H. (2007). Prevalence of iron deficiency and anemia among healthy women of reproductive age in Bhaktapur, Nepal. Eur. J. Clin. Nutr..

[29] Husek P. (1995). Simultaneous profile analysis of plasma amino and organic acids by capillary gas chromatography. J. Chromatogr. B Biomed. Appl..

[30] Moller J., Rasmussen K., Christensen L. (1999). External quality assessment of methylmalonic acid and total homocysteine. Clin. Chem..

[31] Molloy A.M., Scott J.M. (1997). Microbiological assay for serum, plasma, and red cell folate using cryopreserved, microtiter plate method. Methods Enzymol..

[32] Kelleher B.P., Walshe K.G., Scott J.M., O’Broin S.D. (1987). Microbiological assay for vitamin B12 with use of a colistin-sulfate-resistant organism. Clin. Chem..

[33] Hannisdal R., Ueland P.M., Svardal A. (2009). Liquid chromatography-tandem mass spectrometry analysis of folate and folate catabolites in human serum. Clin Chem..

[34] Wfood2 (1996). World Food 2 Computer Software Package.

[35] World Health Organization (2010). Estimating Appropriate Levels of Vitamins and Minerals for Food Fortification Programs: The WHO Intake Monitoring, Assessment and Planning Program (IMAPP).

[36] Greenland S., Pearl J., Robins J.M. (1999). Causal diagrams for epidemiologic research. Epidemiology.

[37] CDC/MMWR (1989). CDC Criteria for Anemia in Children and Child Bearing Aged Women.

[38] World Health Organization (2012). Serum and Red Blood Cell Folate Concentrations for Assessing Folate Status in Population.

[39] Refsum H., Yajnik C.S., Gadkari M., Schneede J., Vollset S.E., Orning L., Guttormsen A.B., Joglekar A., Sayyad M.G., Ulvik A. (2001). Hyperhomocysteinemia and elevated methylmalonic acid indicate a high prevalence of cobalamin deficiency in Asian Indians. Am. J. Clin. Nutr..

[40] Hirsch S., de la Maza P., Barrera G., Gattas V., Petermann M., Bunout D. (2002). The Chilean flour folic acid fortification program reduces serum homocysteine levels and masks vitamin B-12 deficiency in elderly people. J. Nutr..

[41] WHO/FAO (2004). Vitamin and Mineral Requirements in Human Nutrition.

[42] ICMR (2010). Nutrient Requirements and Recommended Dietary Allowances for Indians.

[43] Ulak M., Chandyo R.K., Adhikari R.K., Sharma P.R., Sommerfelt H., Refsum H., Strand T.A. (2014). Cobalamin and folate status in 6 to 35 months old children presenting with acute diarrhea in Bhaktapur, Nepal. PLoS ONE.

[44] Misra A., Vikram N.K., Pandey R.M., Dwivedi M., Ahmad F.U., Luthra K., Jain K., Khanna N., Devi J.R., Sharma R. (2002). Hyperhomocysteinemia, and low intakes of folic acid and vitamin B12 in urban North India. Eur. J. Nutr..

[45] Carmel R., Mallidi P.V., Vinarskiy S., Brar S., Frouhar Z. (2002). Hyperhomocysteinemia and cobalamin deficiency in young Asian Indians in the united states. Am. J. Hematol..

[46] Hung C.J., Huang P.C., Lu S.C., Li Y.H., Huang H.B., Lin B.F., Chang S.J., Chou H.F. (2002). Plasma homocysteine levels in Taiwanese vegetarians are higher than those of omnivores. J. Nutr..

[47] Lindgren A. (2002). Elevated serum methylmalonic acid. How much comes from Cobalamin deficiency and how much comes from the kidneys?. Scand. J. Clin. Lab. Investig..

[48] Joshi D.D., Maharjan M., Johansen M.V., Willingham A.L., Sharma M. (2003). Improving meat inspection and control in resource-poor communities: The Nepal example. Acta Trop..

[49] Albert M.J., Mathan V.I., Baker S.J. (1980). Vitamin B12 synthesis by human small intestinal bacteria. Nature.

[50] Brouwer-Brolsma E.M., Dhonukshe-Rutten R.A., van Wijngaarden J.P., Zwaluw N.L., Velde N., de Groot L.C. (2015). Dietary sources of vitamin B-12 and their association with vitamin B-12 status markers in healthy older adults in the B-PROOF study. Nutrients.

[51] FAO/WHO (2000). Food and Agricultural Organization (FAO) and World Health Organization (WHO) Expert Consultation on Human Vitamin and Mineral Requirements.

[52] Bondevik G.T., Ulstein M., Lie R.T., Rana G., Kvale G. (2000). The prevalence of anemia in pregnant Nepali women—A study in Kathmandu. Acta Obstet. Gynecol. Scand..

[53] Steegers-Theunissen R.P., Boers G.H., Trijbels F.J., Eskes T.K. (1991). Neural-tube defects and derangement of homocysteine metabolism. N. Engl. J. Med..

[54] Scott J.M., Weir D.G. (1981). The methyl folate trap. A physiological response in man to prevent methyl group deficiency in kwashiorkor (methionine deficiency) and an explanation for folic-acid induced exacerbation of subacute combined degeneration in pernicious anemia. Lancet.

[55] Dwarkanath P., Barzilay J.R., Thomas T., Thomas A., Bhat S., Kurpad A.V. (2013). High folate and low vitamin b-12 intakes during pregnancy are associated with small-for-gestational age infants in South Indian women: A prospective observational cohort study. Am. J. Clin. Nutr..

[56] Bjorkegren K., Svardsudd K. (2001). Serum cobalamin, folate, methylmalonic acid and total homocysteine as vitamin B12 and folate tissue deficiency markers amongst elderly swedes—A population-based study. J. Intern. Med..

